# Purification of prothrombin complex proteins from human plasma in anion exchange resin using pseudoaffinity chromatography

**DOI:** 10.1186/1753-6561-8-S4-P23

**Published:** 2014-10-01

**Authors:** Vinicius Watanabe Nakao, Claudia Iwashita Verinaud, Gabriel Feliciano Pinna, André Conti Luiz, Isaías Raw, Elisabeth AL Martins, Elisabeth Cheng

**Affiliations:** 1 Centro de Biotecnologia, Instituto Butantan - Av. Vital Brasil 1500, 05503-090, São Paulo, Brasil

## Background

Prothrombin complex contains the vitamin K dependent coagulation factors II, VII, IX, X, protein C, and protein S. It has been used for the treatment of congenital coagulation disorders and is recommended for reversing oral anticoagulation [1]. Prothrombin complex proteins require Ca^2+ ^to express their activities. The conformational change induced by Ca^2+ ^finds a practical application in the purification processes by modifying the affinity of these proteins to chromatographic resins. The elution of proteins by variation of calcium concentration is called chromatography of pseudoaffinity [2]. In this study we exploit this property of the vitamin dependent coagulation factors to develop a new method for purification of prothrombin complex proteins from human plasma using an anion exchange resin.

## Methods

Plasma was directly applied to the ANX Sepharose FF column, previously equilibrated with citrate buffer 25 mM containing NaCl 85 mM and CaCl_2_, pH 6. The unbound proteins were washed out with the same buffer. After a washing with citrate buffer containing NaCl 200 mM, elution was carried out in the same buffer with a linear calcium gradient from 2.5 mM to 25 mM. Three different buffers were tested: citrate, Bis-Tris and MES. Finally, column was washed with citrate buffer containing 500 mM NaCl. Chromatographic fractions were analyzed by: activity of Protein C using the chromogenic method as representative of the prothrombin complex proteins, protein content by the Bradford method, and SDS-PAGE.

## Results and discussion

Protein C eluted within the CaCl_2 _concentration range studied (2.5 to 25 mM). Using NaCl, this protein eluted only with a much higher salt concentration (> 250 mM), confirming that the mechanisms of elution with these 2 salts are different. It was also observed that a wash with 200 mM NaCl improved the purification. Therefore, the method combines conventional anion exchange with pseudoaffinity chromatography. Chromatograms of the experiments presented different profiles: citrate buffer presented 2 peaks, while Bis-Tris and MES presented only one, indicating that citrate buffer led to a better separation of the proteins. The SDS-PAGE gels showed that contaminant proteins coeluted with the prothrombin complex proteins, but in comparison to purifications with NaCl gradient, a much better purification was achieved with the CaCl_2 _gradient. Further experiments will be performed to identify the CaCl_2 _eluted proteins.
